# Construction of a modified TNM staging system and prediction model based on examined lymph node counts for gastric cancer patients at pathological stage N3

**DOI:** 10.3389/fonc.2025.1569736

**Published:** 2025-04-03

**Authors:** Hongyu Zhang, Nan Sun, Feng Li, Qiyang Wang, Zhao Sun, Yawei Zhang, Lei Wang, Chunlin Zhao, Yang Fu

**Affiliations:** ^1^ Department of Gastrointestinal Surgery, The First Affiliated Hospital of Zhengzhou University, Zhengzhou, China; ^2^ Department of Plastic Surgery, The First Affiliated Hospital of Zhengzhou University, Zhengzhou, China; ^3^ Department of Thoracic Surgery, The First Affiliated Hospital of Zhengzhou University, Zhengzhou, China; ^4^ Department of Pathology, The First Affiliated Hospital of Zhengzhou University, Zhengzhou, China

**Keywords:** N3 gastric cancer, examined lymph nodes, modified TNM staging system, survival, prediction model

## Abstract

**Background:**

Examined lymph node (ELN) count is a critical factor affecting the number of metastatic lymph nodes (MLNs). The impact of the ELN number on survival and staging remains unclear.

**Methods:**

This study included 4,291 stage N3 GC patients from the SEER database (training cohort) and 567 stage N3 GC patients from the FAHZZU database (validation cohort). The optimal ELN count and stage migration were investigated, and a modified TNM (mTNM) staging system including the ELN count was proposed. LASSO regression and random forest analyses were used to screen and evaluate the variables associated with survival, and an mTNM-based nomogram was constructed. The performance of the mTNM staging system and mTNM-based nomogram were compared with that of the 8th edition of the TNM staging system.

**Results:**

The optimal threshold of the ELN count was identified as 21. An insufficient number of ELNs (≤ 21) was associated with poorer survival outcomes and led to stage migration in all N3 patients. A new mTNM staging system was proposed, integrating the ELN count into the TNM staging system (8th edition). LASSO regression analysis revealed that age, tumor size, adjuvant chemotherapy, adjuvant radiotherapy, and the mTNM system were associated with overall survival (OS) outcomes, and random forest analysis revealed that the mTNM system was the most important variable for predicting survival. An mTNM-based nomogram was constructed to predict 1-, 3-, and 5-year OS rates. Compared with the TNM staging system (8th edition), the mTNM staging system and mTNM-based nomogram showed superior prognosis discriminative ability, better predictive accuracy, and greater net improvement in survival outcomes.

**Conclusions:**

The optimal ELN count for N3 GC patients was 21. The mTNM staging system and mTNM-based nomogram showed superior discriminative ability, predictive accuracy, and greater net benefit for OS outcomes.

## Introduction

Gastric cancer (GC) is the fifth most common malignant tumor and the fourth leading cause of cancer-related death worldwide (1). Despite the availability of a multidisciplinary approach, the prognoses of patients with advanced-stage GC remain poor (2). Lymph node (LN) involvement is one of the most important prognostic factors within the first 5 years after curative gastrectomy (3, 4). Therefore, a credible and practical staging system based on LN involvement is crucial for the treatment and prognostic prediction of GC. Although the 8th edition of the Union for International Cancer Control (UICC)/American Joint Commission on Cancer (AJCC) tumor–node–metastasis (TNM) staging system is currently the most widely used staging system, the reliability of its LN staging has been controversial (4, 5). Several studies have demonstrated that the LN staging of the UICC/AJCC TNM system, which is based on the number of metastatic lymph nodes (MLNs), is influenced by the number of examined LNs (ELNs) and likely leads to stage migration (5–7). This system asserts that retrieving at least 16 LNs after curative surgery is essential for reasonable staging (8). However, many studies have demonstrated that 16 ELNs might be insufficient to ensure reliable staging, especially in advanced GC patients (7, 9, 10). Stage N3, which includes patients with ≥ 7 MLNs (N3a: 7–15 MLNs; N3b: ≥ 16 MLNs), is mainly classified as stage III according to the 8th edition of the TNM staging system. Stage N3 patients comprise a considerable proportion of all GC patients, especially East Asian patients (11, 12). In general, the prognoses of stage N3 GC patients are very poor even after curative procedures (12). Assessing fewer than 16 ELNs cannot detect N3b patients and could lead to inaccurate staging and unreliable survival predictions. Moreover, inaccurate staging may impact decision-making regarding adjuvant treatment after identifying a potential adverse prognosis, as adjuvant chemotherapy and chemoradiotherapy are indispensable in improving the survival of patients with advanced GC (13–15). The optimal cutoff value of the ELN count for stage N3 GC patients remains uncertain, as does whether considering the ELN and MLN counts simultaneously in the staging system could address the issue of stage migration. In this study, we identified the optimal number of ELNs for stage N3 GC patients and proposed a modified TNM (mTNM) staging system that integrates the ELN count. We also constructed an mTNM-based nomogram to predict the 1-, 3-, and 5-year OS outcomes of stage N3 GC patients and compared the performance of the mTNM staging system and mTNM-based nomogram to that of the 8th edition of the TNM staging system.

## Materials and methods

### Population

Training cohort (SEER data): Stage N3 (MLNs ≥ 7) GC patients were screened from the Surveillance, Epidemiology, and End Results (SEER) database (Primary Site-labeled: C16.0–C16.9) via SEER*Stat software (version 8.4.2). We were authorized to use SEER data without the need for local ethical approval or declaration. The inclusion criteria were as follows: aged 18–80 years, histologically confirmed gastric carcinoma diagnosed between 2004–2020, pathological stage T1–4N3M0 GC (≥ 7 MLNs), and overall survival exceeding one month after gastrectomy. The exclusion criteria were as follows: a history of other malignancies and insufficient pathological staging or follow-up information.

The validation cohort (First Affiliated Hospital of Zhengzhou University (FAHZZU) database) included stage N3 (MLNs ≥ 7) GC patients whose data were collected from electronic medical records at the FAHZZU and retrospectively collected data from pathologically diagnosed GC patients who underwent gastrectomy from January 2016 to December 2020. This study was approved by the Ethics Committee of the First Affiliated Hospital of Zhengzhou University (2023-KY-0913-003), and the requirement for individual consent for this retrospective analysis was waived. The inclusion criteria were as follows: aged 18–80 years, pathological stage T1–4N3M0 GC (≥ 7 MLNs), histologically confirmed R0 resection (defined as no macroscopic or microscopic residual tumor), no distant metastases or gastric stump cancer, and no other malignant tumors. The exclusion criteria mentioned above for the SEER data were also applied to the FAHZZU data. After curative surgery, patients were followed every 3 months for the first 2 years, every 6 months for the next 3 years, and annually thereafter. The follow-up terms included physical examination, gastrointestinal (GI) tumor markers, contrast-enhanced computed tomography (CT; chest, abdomen, and pelvis), and upper-GI endoscopy annually or when clinically indicated. We concluded follow-ups of all enrolled patients in September 2022. The median follow-up time was 21 (IQR, 12–36) months.

The endpoint was overall survival (OS; months), defined as the time from the date of curative surgery to the date of death from any cause. Data for patients who did not reach this endpoint by the date of their last follow-up visit were regarded as censored. This study was performed in compliance with the Declaration of Helsinki (16). We prospectively registered this study in the Chinese Clinical Trial Registry (ChiCTR). The registration number was ChiCTR2400084628.

### Clinicopathological variables

Patient demographics, pathological variables, survival time, adjuvant chemotherapy, and adjuvant radiotherapy information were extracted from the SEER and FAHZZU databases. Clinicopathological variables were as follows: (1) patient demographics (age, sex, race); (2) pathological variables (tumor location, tumor size [long diameter], histology, Lauren’s type, pathological T stage, pathological N stage, pathological TNM stage [UICC/AJCC TNM staging system, 8th edition], and ELN count); (3) adjuvant chemotherapy and radiotherapy information; and (4) overall survival and survival status at the last follow-up.

### Cutoff values for the ELN count and development of a modified TNM staging system and nomogram prediction model

The cutoff value of the ELN count was explored according to the largest statistics of the log-rank test in the training and validation cohorts. Kaplan–Meier survival analysis was employed to evaluate the stage migration caused by the ELN count. A modified TNM (mTNM) staging system was proposed by integrating ELN count into the TNM staging system (8th edition). Least absolute shrinkage and selection operator (LASSO) regression was used to screen variables significantly associated with survival, and random survival forest (RSF) analysis was performed to assess variable importance. The screened variables were incorporated to construct a prognostic nomogram (mTNM-based nomogram) for the prediction of 1-, 3-, and 5-year OS outcomes. We subsequently compared the discriminative ability, predictive ability, and clinical usefulness of the TNM staging system (8th edition), the mTNM staging system, and the mTNM-based nomogram prediction model.

### Statistical analysis

The cutoff value of the ELN count was explored via the survminer package (https://CRAN.R-project.org/package=survminer) in R software (version 4.3.2), which determines the optimal cutoff point for one or multiple continuous variables simultaneously, uses the maximally selected rank statistics, and provides a cutoff value that corresponds to the most significant relationship with survival. The tumor size and age variables were also transformed into categorical variables according to the largest log-rank value calculated via the survminer package. The correlation between the number of ELNs and the number of MLNs was analyzed via Spearman’s test. Least absolute shrinkage and selection operator (LASSO) regression, which is based on the Cox model (LASSO–Cox regression), was used to screen variables by minimizing the prediction error and penalizing the absolute size of the regression coefficients (17). Lambda.1se was regarded as the optimal lambda (λ) for screening variables and was used to construct a prediction model. An RSF analysis was also performed to assess the importance of the variables through survival trees. RSF analysis involves the construction of numerous survival trees via the bootstrap method, which ensures that each tree is grouped into distinct data samples. The RSF algorithm inherently calculates the importance score (variable importance, VIMP) contributed by each variable to the model’s predictive accuracy. A positive VIMP score indicates that the variable can increase the accuracy of the prediction model, whereas a negative VIMP score indicates that the variable does not help predict accuracy. Multivariate Cox regression analysis was used to assess the role of the screened variables as prognostic factors with hazard ratios (HRs) and a 95% confidence interval (CI). Survival curves were drawn via the Kaplan–Meier method and compared via the log-rank test. The Harrell’s concordance index (C-index) was calculated, and 1-, 3-, and 5-year time-dependent ROC curves were drawn to assess and compare the discriminative abilities of the TNM staging system (8th edition), mTNM staging system and mTNM-based nomogram, with higher C-indexes and larger areas under the ROC curves (AUCs) indicating a better model for survival discrimination. Calibration curves were plotted to compare the agreement between the predicted survival probabilities and the actual outcome frequencies; the closer the distance between the ideal curve and the predicted curve was, the more accurate the actual prediction ability was. Decision curve analysis (DCA) curves were used to determine the clinical utility of each model by assessing the net benefits at different threshold probabilities. When the net benefit rate corresponding to a probability threshold is located on the upper right side of the All line and None line, the predictive model has good clinical utility. We used R software version 4.3.2 to analyze all the data. The R packages utilized in this study include: tidyverse, export, ggsci, survminer, survival, rms, pec, riskRegression, and dcurves. A two-sided *P* value < 0.05 was considered to indicate significance.

## Results

### General characteristics

**Figure 1:**
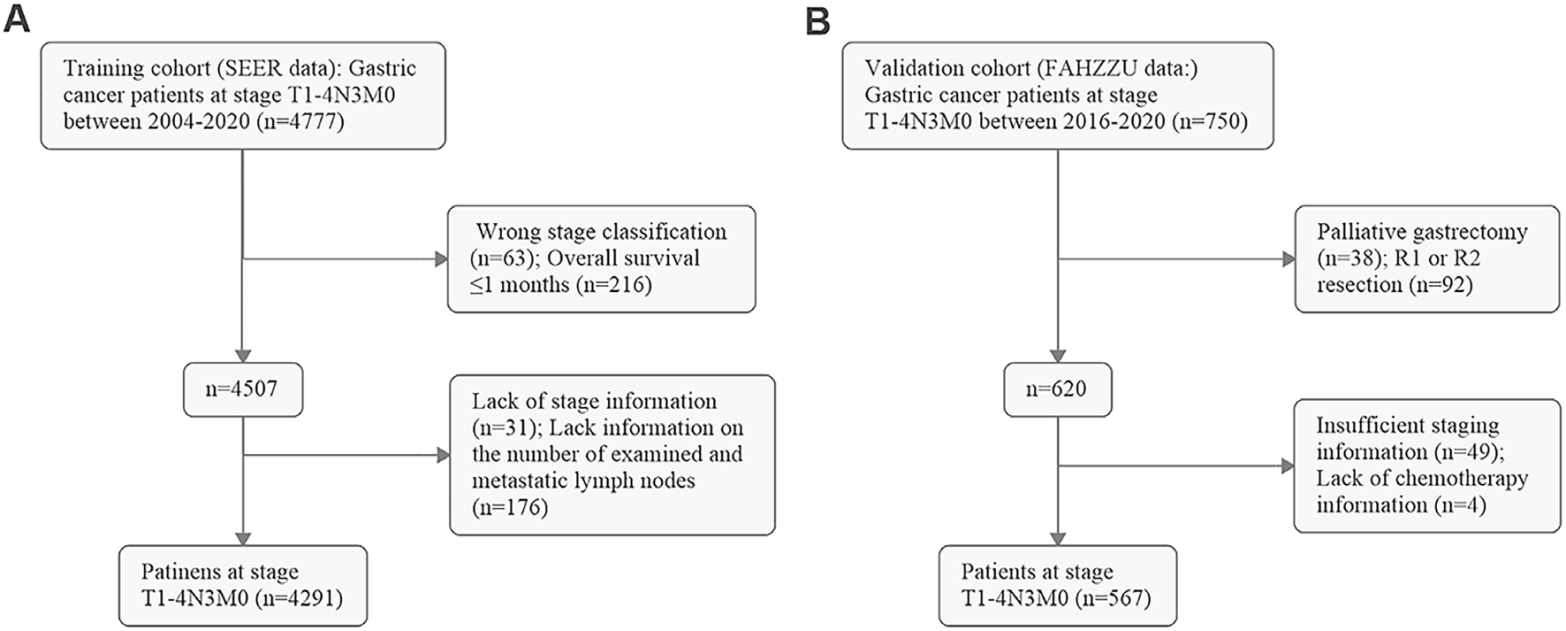
Flow diagram of screening patients in **(A)** the training cohort (SEER data) and **(B)** the validation cohort (FAHZZU data).

**Figure 2:**
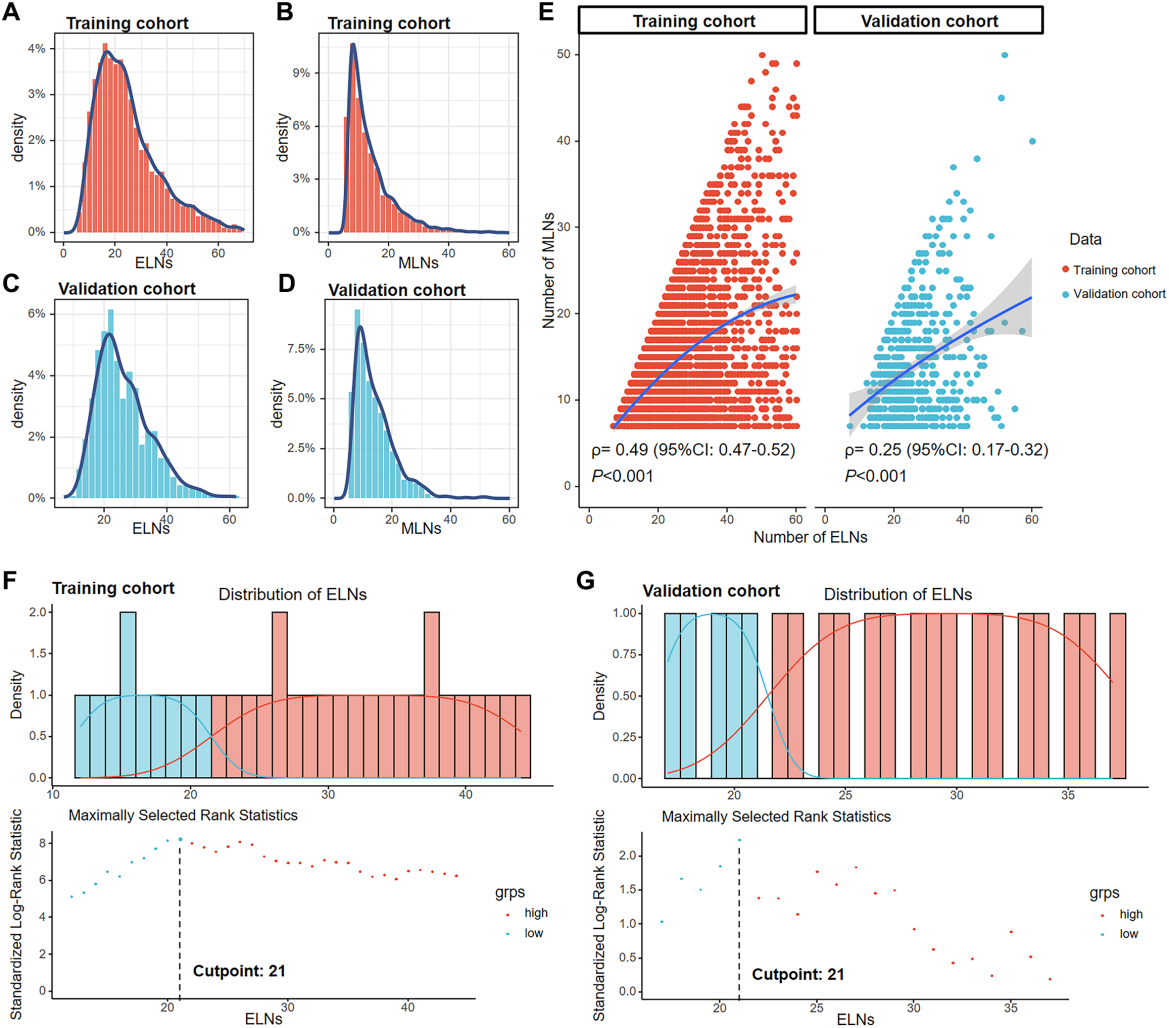
Distribution of ELNs and MLNs in the training cohort **(A, B)** and the validation cohort **(C, D)**. Correlations of the number of MLNs with the number of ELNs in the training and validation cohorts according to Spearman’s test **(E)**. The best cutoff point for ELNs was 21, according to the maximally standardized log-rank statistic in the training **(F)** and validation **(G)** cohorts.

After screening, 4,291 GC patients in the training cohort (SEER data) and 567 GC patients in the validation cohort (FAHZZU data) were ultimately enrolled in the study (**Figure 1**). The clinicopathological characteristics of these patients are summarized in **Table 1**. The 1-, 3-, and 5-year OS rates of the two cohorts were 66.5, 27.2, and 18.0% and 77.1, 40.0, and 30.3%. The mean and median ELNs were 26.0 (sd. 14.4) and 23 (range 7–98) in the training cohort and 26.3 (sd. 8.7) and 24 (range 7–62) in the validation cohort. Approximately 70.1 and 29.9% of the patients in the training cohort and 67.5 and 32.5% of those in the validation cohort, were N3a and N3b, respectively. The ELNs and MLNs in the training cohort (**Figures 2A, B**) and validation cohort (**Figures 2C, D**) were all positively skewed. Spearman’s test revealed that the number of MLNs was positively correlated with the number of ELNs in both the training cohort (ρ= 0.49, *P* < 0.001) and the validation cohort (ρ = 0.25, *P* < 0.001) (**Figure 2E**).

**Table 1:** General characteristics of N3 GC patients in the training cohort and validation cohort.

Variables	Training cohort (n = 4291)	Validation cohort (n = 567)	χ^2^	*P*
**Age**			11.598	<0.001
≤60	1814 (42.3%)	283 (49.9%)		
>60	2477 (57.7%)	284 (50.1%)		
**Sex**			33.701	<0.001
Female	1630 (38%)	144 (25.4%)		
Male	2661 (62%)	423 (74.6%)		
**Race**			1421.9	<0.001
W	2743 (63.9%)	0		
B	560 (13.1%)	0		
AI	44 (1.0%)	0		
API	944 (22.0%)	567 (100%)		
**Tumor location**			178.82	<0.001
Cardia/fundus	957 (22.3%)	237 (41.8%)		
Body	435 (10.1%)	63 (11.1%)		
Antrum/pylorus	1275 (29.7%)	199 (35.1%)		
Others	1624 (37.8%)	68 (12%)		
**Tumor size**			30.573	<0.001
<8 cm	3074 (71.6%)	469 (82.7%)		
≥8 cm	1217 (28.4%)	98 (17.3%)		
**Grade**			22.857	<0.001
Well	39 (0.9)	0		
Moderate	550 (12.8)	82 (14.5)		
Poor	3578 (83.4)	485 (85.5)		
Undifferentiated	124 (2.9)	0		
**Histology**			119.61	<0.001
Adenocarcinoma	2449 (57.1%)	454 (80.1%)		
SRCC	1287 (30%)	100 (17.6%)		
Others	555 (12.9%)	13 (2.3%)		
**Lauren′s type**			635.38	<0.001
Intestinal	451 (10.5%)	106 (18.7%)		
Diffuse	486 (11.3%)	181 (31.9%)		
Mixed	197 (4.6%)	137 (24.2%)		
Others	3157 (73.6%)	143 (25.2%)		
**Adjuvant chemotherapy**			8.879	0.003
No	1058 (24.7%)	107 (18.9%)		
Yes	3233 (75.3%)	460 (81.1%)		
**Adjuvant radiotherapy**			458.62	<0.001
No	2226 (51.9%)	563 (99.3%)		
Yes	2065 (48.1%)	4 (0.7%)		
**ELNs**	26.0 ± 14.4* 23 (7–98)^#^	26.3 ± 8.7* 24 (7–62)^#^	–	–
**MLNs**	14.1 ± 8.1* 12 (7-84)^#^	14.0 ± 6.7* 12 (7-52)^#^	–	–
**T stage**			224.72	<0.001
T1	109 (2.5%)	6 (1.1%)		
T2	190 (4.4%)	39 (6.9%)		
T3	1835 (42.8%)	418 (73.7%)		
T4a	1696 (39.5%)	77 (13.6%)		
T4b	461 (10.7%)	27 (4.8%)		
**N stage**			1.374	0.241
N3a	3006 (70.1%)	383 (67.5%)		
N3b	1285 (29.9%)	184 (32.5%)		
**TNM stage**			8.837	0.032
IIB	88 (2.1%)	4 (0.7%)		
IIIA	155 (3.6%)	30 (5.3%)		
IIIB	2522 (58.8%)	339 (59.8%)		
IIIC	1526 (35.6%)	194 (34.2%)		

*Mean ± SD; ^#^Median (range); W, White; B, Black; AI, American Indian; API, Asian or Pacific Islander; SRCC, signet ring cell carcinoma; ELNs, examined lymph nodes; MLNs, metastatic lymph nodes.

### Cutoff of the ELN count

In the training cohort, the numbers of ELNs corresponding to the top three log-rank statistics were 21, 20, and 26, respectively (**Figure 2F**). In the validation cohort, the numbers of ELNs corresponding to the top three log-rank statistics was 21, 20 and 27, respectively (**Figure 2G**). The largest log-rank statistic indicates the most significant difference in survival between the two groups. Therefore, 21 was selected as the best cutoff value for ELNs in both the training and validation cohorts. We subsequently divided patients into ≤ 21-ELN and > 21-ELN groups. The general characteristics of the training and validation cohorts grouped by ELN count are shown in **Supplementary Table S1** and **Supplementary Table S2**, respectively. Patients in the ≤ 21-ELN subgroup had a greater probability of being grouped into the N3a subgroup than patients in the > 21-ELN subgroup did (training cohort: 90.6 *vs.* 52.7%, *P* < 0.001; validation cohort: 84.7 *vs.* 59.0%, *P* < 0.001). As a result, patients with ELNs≤21 are also more likely to be grouped into the early TNM stage, and a greater proportion of these patients are classified into the IIIB stage than patients in the > 21-ELN group (training cohort: 74.1 *vs.* 45.7%; validation cohort: 77.2 *vs.* 51.1%). In the IIIC stage, only 25.6% (390/1526) of patients were in the ≤ 21-ELN group and 74.4% (1136/1526) were in the > 21-ELN group. The stage was clearly influenced by the ELN count.

### Stage migration and construction of the modified TNM staging system

**Figure 3:**
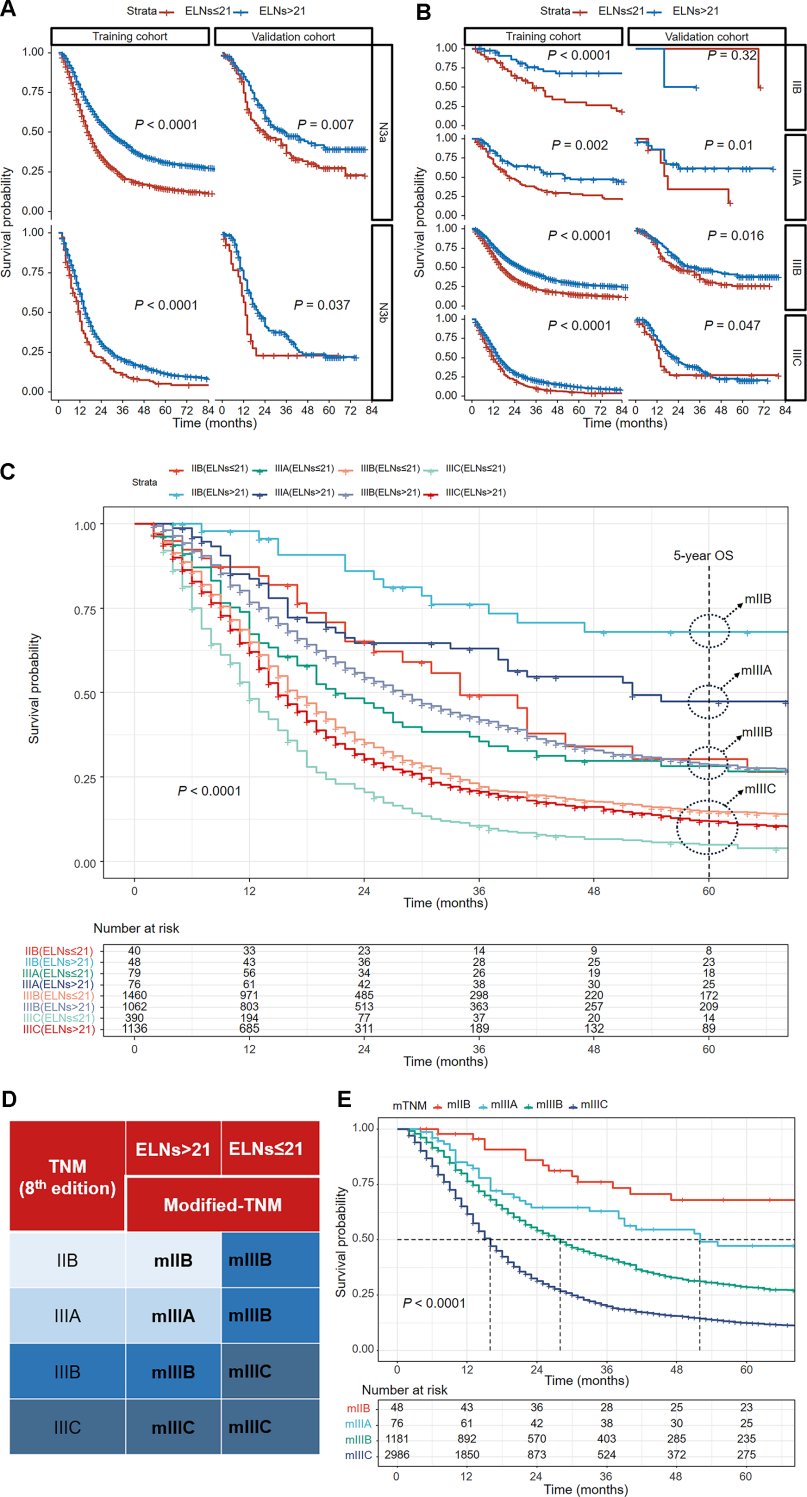
Kaplan–Meier curves comparing overall survival between the ≤ 21-ELN group and the > 21-ELN group in the training and validation cohorts for N3a and N3b patients **(A)** and for IIB, IIIA, IIIB, and IIIC patients **(B)**. Kaplan–Meier curves showing overall survival comparisons of eight subgroups grouped by the ELNs in the TNM staging system (8th edition) in the training cohort **(C)**. The groups with similar 5-year overall survival estimations were reclassified into a new group. The modified TNM system was established on the basis of the combination of ELNs and the TNM staging system (8th edition) **(D)**. Kaplan–Meier curves for overall survival of patients with different pathological stages according to the new modified TNM staging system **(E)**.

Among N3 patients, those with insufficient ELNs (≤ 21) exhibited poorer OS outcomes than those with > 21 ELNs in both the N3a and N3b stages (**Figure 3A**). When the training cohort was stratified by the TNM staging system (8th edition), the ≤ 21-ELN subgroup presented significantly poorer OS than did the > 21-ELN subgroup in stages IIB, IIIA, IIIB, and IIIC (**Figure 3B**). In the validation cohort, except for no statistically significant OS outcomes in IIB stage patients, poorer survival outcomes were presented in the ≤ 21-ELN group for IIIA, IIIB, and IIIC patients according to the Kaplan–Meier curves (**Figure 3B**). Therefore, for the same pathological stage classified by the 8th edition of the TNM system, OS rates are not homogeneous. Insufficient ELNs (≤ 21) were associated with poorer OS outcomes, leading to stage migration in N3 GC patients.

Considering the stage migration caused by ELNs, we intended to modify the TNM staging system (8th edition) by integrating ELN as a factor. On the basis of the 5-year OS estimations of IIB–IIIC patients grouped by ELN count (**Table 2**), patients with similar outcomes were reclassified into a new group, and a modified TNM (mTNM) staging system was constructed (**Figure 3C**). In the new mTNM system, the mIIB stage includes IIB patients with ELNs > 21; the mIIIA stage includes IIIA patients with ELNs > 21; the mIIIB stage includes IIIB patients with ELNs > 21, IIB patients with ELNs ≤ 21, and IIIA patients with ELNs ≤ 21; and the mIIIC stage includes IIIC patients and IIIB patients with ELNs ≤ 21 (**Figure 3D**). The Kaplan–Meier curves in the new mTNM staging system were used to estimate the survival of each group (**Figure 3E**). The 5-year OS rates of each group are shown in **Table 3**.

**Table 2:** The 5-year OS outcomes of the 8 subgroups grouped by ELN count according to the TNM staging system (8th edition).

Subgroups	5-year OS (95% CI)	Subgroups	5-year OS (95% CI)
**IIB (ELNs≤21)**	30.3% (17.5%–52.4%)	**IIB (ELNs>21)**	68.0% (54.9%–84.1%)
**IIIA (ELNs≤21)**	28.2% (19.5%–40.8%)	**IIIA (ELNs>21)**	47.3% (36.4%–61.4%)
**IIIB (ELNs≤21)**	14.7% (12.9%–16.7%)	**IIIB (ELNs>21)**	28.5% (25.7%–31.7%)
**IIIC (ELNs≤21)**	4.9% (3.0%–7.8%)	**IIIC (ELNs>21)**	11.9% (10.0%–14.2%)

**Table 3:** 5-year OS of the modified TNM staging system.

Modified-TNM staging system (mTNM)		5-year OS (95% CI)
**mIIB**	IIB (ELNs>21)	68.0% (54.9%–84.1%)
**mIIIA**	IIIA (ELNs>21)	47.3% (36.4%–61.4%)
**mIIIB**	IIB (ELNs≤21), IIIA (ELNs≤21), IIIB (ELNs>21)	28.6% (25.9%–31.6%)
**mIIIC**	IIIB (ELNs≤21), IIIC (ELNs>21), IIIC (ELNs≤21)	12.4% (11.2%–13.8%)

mTNM, modified TNM staging system.

### Development of the mTNM-based nomogram

**Figure 4:**
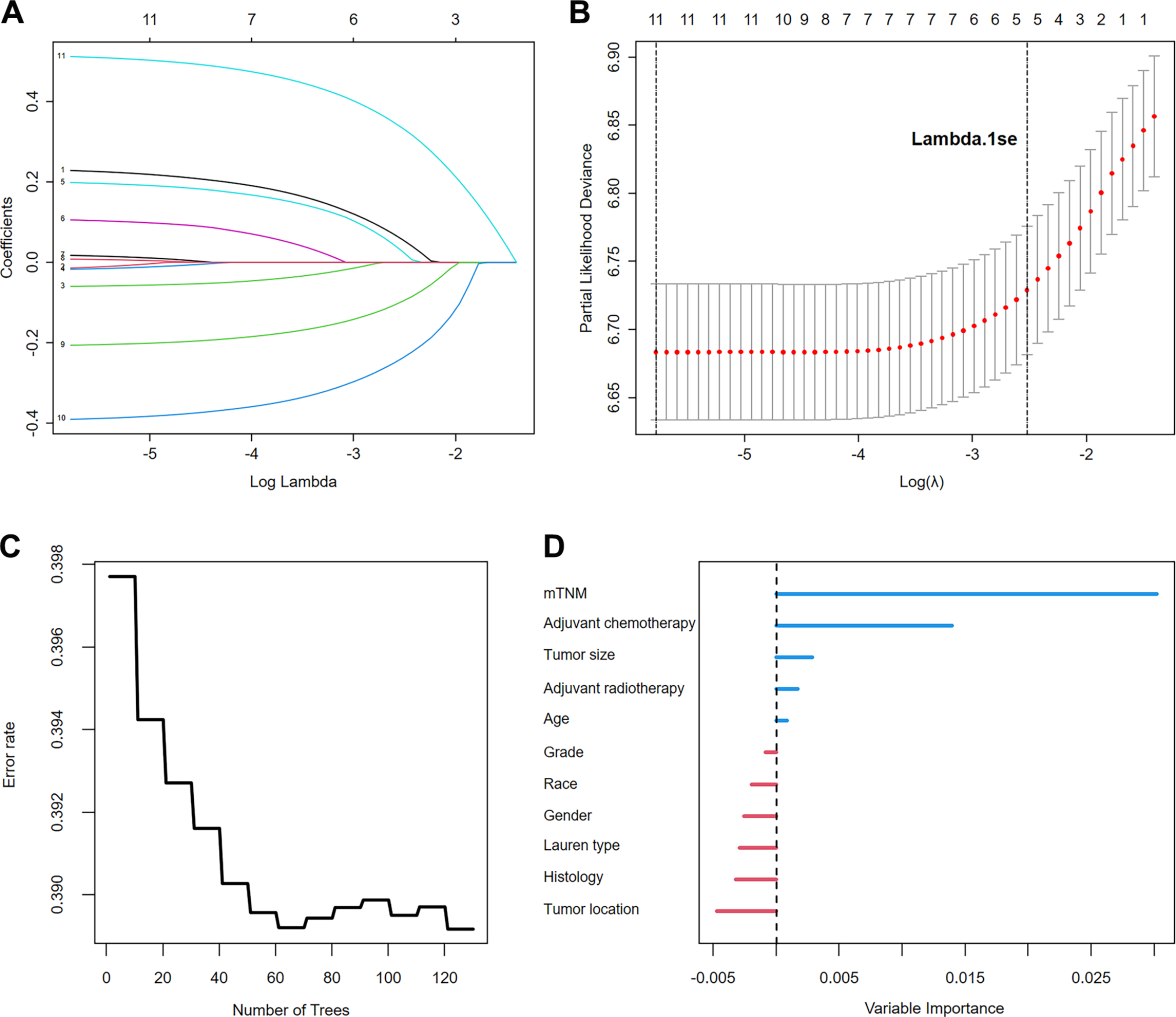
Variable screening using LASSO regression analysis: **(A)**. LASSO regression coefficient path; **(B)**. Cross-validation for optimal λ selection. The random survival forest identified the importance of the variables: **(C)**. Error rate of the random survival forest; **(D)**. Importance ranking of variables.

**Figure 5:**
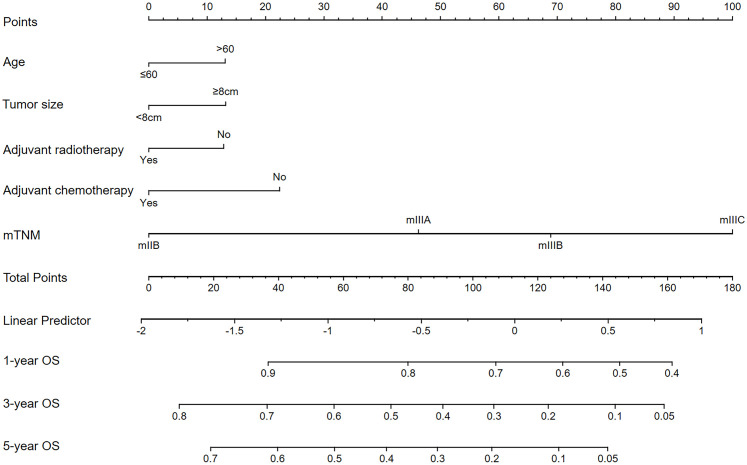
The mTNM-based nomogram for predicting the 1-, 3-, and 5-year OS outcomes of stage N3 GC patients.

LASSO–Cox regression was performed to screen variables associated with OS outcomes. The analyzed variables included age, sex, race, tumor location, tumor size, grade, histology, Lauren’s type, adjuvant chemotherapy, adjuvant radiotherapy, and the mTNM staging system (including factors such as T stage, N stage, M stage, and ELNs [cutoff value: 21]). The curve of the regression coefficient path was used to screen the variables (**Figure 4A**). The 10-fold cross-validation method was used for the interactive analysis, and a model with excellent performance but a minimum number of variables was obtained when lambda.1se (λ.1se) was 0.08037415 (log λ= −2.541063) (**Figure 4B**). The screened variables were age, tumor size, adjuvant chemotherapy, adjuvant radiotherapy, and the mTNM staging system. In the RSF analysis, the model’s error rate stabilized when 130 trees were constructed (**Figure 4C**). The variables associated with positive VIMP scores were age, tumor size, adjuvant chemotherapy, adjuvant radiotherapy, and the mTNM staging system (**Figure 4D**). The variable with the highest positive VIMP score was the mTNM staging system, indicating that the mTNM staging system was the most important factor for predicting survival with the model. Furthermore, in the multivariate Cox regression analysis, age, tumor size, adjuvant chemotherapy, adjuvant radiotherapy, and the mTNM system were found to be significant independent factors (**Table 4**). Given the above findings, we constructed an mTNM-based prediction model consisting of age, tumor size, adjuvant chemotherapy, adjuvant radiotherapy, and the mTNM staging system to predict the 1-, 3-, and 5-year OS outcomes. A corresponding mTNM-based nomogram was thereby constructed (**Figure 5**).

**Table 4:** Multivariate analysis of the screened variables in the mTNM-based prediction model.

Variables	n (%)	HR (95% CI)	*P*
Age			
≤60	1814 (42.3%)	Reference	
>60	2477 (57.7%)	1.26 (1.17–1.35)	<0.001
Tumor size			
<8 cm	3074 (71.6%)	Reference	
≥8 cm	1217 (28.4%)	1.26 (1.17–1.35)	<0.001
Adjuvant chemotherapy			
No	1058 (24.7%)	Reference	
Yes	3233 (75.3%)	0.68 (0.62–0.74)	<0.001
Adjuvant radiotherapy			
No	2226 (51.9%)	Reference	
Yes	2065 (48.1%)	0.80 (0.74–0.86)	<0.001
mTNM			
mIIB	48 (1.1%)	Reference	
mIIIA	76 (1.8%)	2.29 (1.24–4.20)	0.008
mIIIB	1181 (27.5%)	3.39 (2.00–5.75)	<0.001
mIIIC	2986 (69.6%)	5.82 (3.44–9.85)	<0.001

mTNM, modified TNM staging system.

### Clinical performance of the mTNM staging system and mTNM-based nomogram

**Figure 6:**
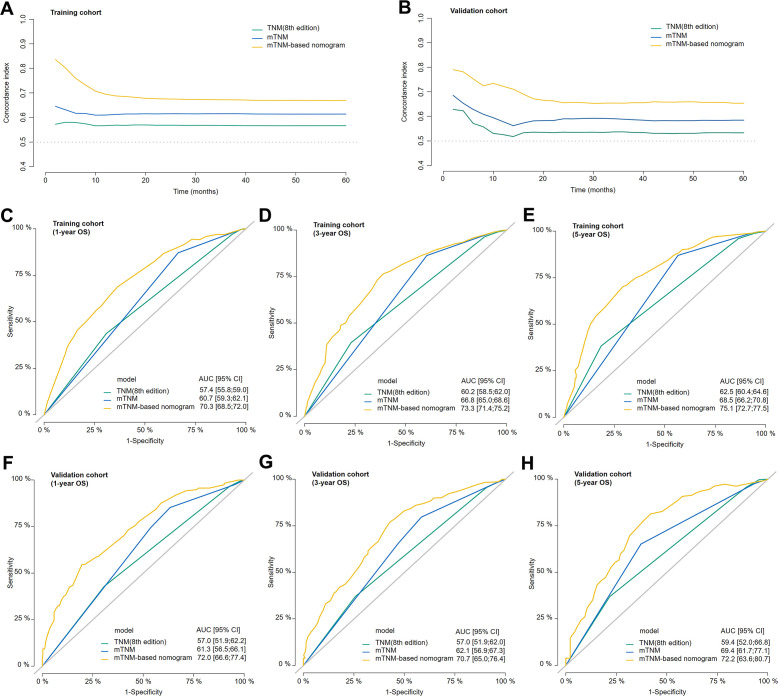
The concordance index (C-index) of the TNM staging system (8th edition), mTNM staging system, and mTNM-based nomogram in the training cohort **(A)** and validation cohort **(B)**. Time-dependent ROC curves showing the predictive power of the mTNM-based nomogram compared with the TNM staging system (8th edition) and the mTNM staging system for the 1-year **(C)**, 3-year **(D)**, and 5-year **(E)** OS outcomes in the training cohort and the 1-year **(F)**, 3-year **(G)** and 5-year **(H)** OS outcomes in the validation cohort.

Compared with the TNM staging system (8th edition), the mTNM staging system had a greater C-index over 5 years in both the training and validation cohorts (**Figures 6A, B**). The mTNM-based nomogram had a higher C-index than these two staging systems did. The AUCs of the 1-, 3-, and 5-year OS rates for the mTNM-based nomogram were 0.703, 0.733, and 0.751, respectively, which were all greater than the AUCs for the mTNM staging system, followed by the TNM staging system (8th edition) in both the training cohort (**Figures 6C–E**) and the validation cohort (**Figures 6F–H**). These results suggest that in terms of survival prediction ability, the mTNM-based nomogram is superior to the mTNM staging system, whereas the mTNM staging system is superior to the TNM staging system (8th edition).

**Figure 7:**
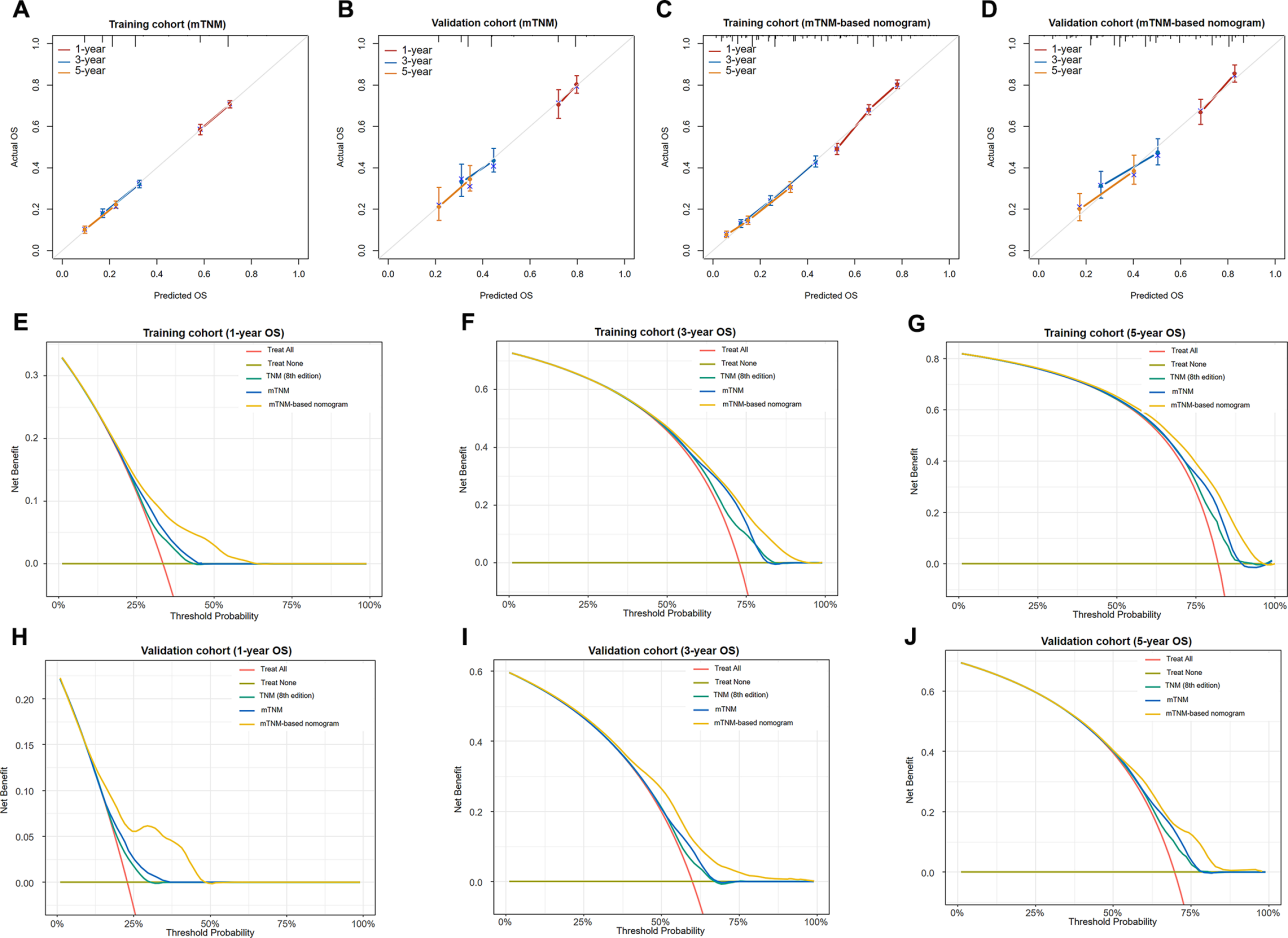
Calibration curves of the mTNM staging system for 1-, 3-, and 5-year OS outcomes in the training cohort **(A)** and the validation cohort **(B)**; calibration curves of the mTNM-based nomogram for the 1-, 3-, and 5-year OS outcomes in the training cohort **(C)** and the validation cohort **(D)**. Decision curve analysis (DCA) of the TNM staging system (8th edition), mTNM staging system, and mTNM-based nomogram for the 1-year **(E)**, 3-year **(F)**, and 5-year OS outcomes **(G)** in the training cohort and the 1-year **(H)**, 3-year **(I)**, and 5-year OS outcomes **(J)** in the validation cohort.

The calibration curves were then plotted to evaluate the prediction accuracy by comparing the predicted probabilities against the actual observed outcomes. Regarding the mTNM staging system, the 1-, 3-, and 5-year calibration curves in the training (**Figure 7A**) and validation (**Figure 7B**) cohort were relatively close to the ideal curves, suggesting that the mTNM staging system has high predictive accuracy. Similarly, regarding the mTNM-based nomogram, the 1-, 3-, and 5-year calibration curves in the training (**Figure 7C**) and validation (**Figure 7D**) cohort also showed better agreement between the predicted and actual probabilities.

The DCA of the training (**Figures 7E–G**) and validation (**Figures 7H–J**) cohorts for the 1-, 3-, and 5-year OS outcomes revealed that within a certain threshold probability range, the mTNM staging system showed greater net prognostic benefit than did the TNM staging system (8th edition), and the mTNM-based nomogram had greater net prognostic benefit than did the mTNM staging system. In other words, the probability of patients benefiting from the mTNM-based nomogram or mTNM staging system was greater than that of patients benefiting from the TNM staging system (8th edition); however, the mTNM-based nomogram demonstrated the best clinical utility.

## Discussion

LNM plays a vital role in the prognosis of GC patients who have undergone radical gastrectomy (18, 19). The ELN count has been confirmed as a key factor affecting LNM (20, 21). Several studies have shown that a greater number of ELNs is correlated with a better prognosis (6, 9, 22). However, the current definition of the N stage depends only on the MLN count and does not consider the ELN count (8). The latest edition of the UICC/AJCC’s TNM classification of GC defines stage N3 as the presence of ≥ 7 MLNs, with no requirement for the number of ELNs. Owing to a heavy metastatic node burden, stage N3 GC patients have extremely poor prognoses (23). GC patients in this stage have a 5-year OS rate as low as 7.1–20%, whereas the 5-year OS rate for M1 GC patients is 7.6% (24, 25). Bhandare et al. suggested that stage N3 GC should be considered a separate subgroup in which more aggressive treatment strategies should be evaluated and performed (26).

Insufficient ELN numbers are considered a potential risk factor for recurrence in GC patients (27). The guidelines recommend the minimal goal of 16 ELNs after curative surgery; however, this goal appears insufficient, especially in patients with advanced GC (8, 11, 28). In our study, on the basis of the analysis of 4,291 stage N3 GC patients from the training cohort, more than 21 ELNs could lead to better OS outcomes than ≤ 21 ELNs at each stage, and the survival benefit was confirmed in the validation cohort. The OS rate was not homogeneous across patients at the same pathological stage with ≤ 21-ELN and > 21-ELN counts; a phenomenon called “Will Rogers” in human cancers, referring to stage migration, was present in these N3 GC patients (29). The pathological GC stage was underestimated because of an insufficient ELN count. The possible reasons are that MLNs are ignored during examination or that inadequate lymphadenectomy is performed in some patients, which leads to fewer MLNs and stage migration (30). Our study revealed that the number of MLNs was positively correlated with the number of ELNs. Fewer ELNs might reflect low-quality lymphadenectomy, leading to a high possibility of residual tumors and a low rate of cure in gastrectomy patients (18). Some patients with low ELN counts might have had greater potential numbers of positive LNs, but too few LNs were examined or dissected, resulting in early staging but poor survival outcomes. NCCN guidelines for gastric cancer recommend that at least 16 regional LNs be pathologically assessed and that the removal of more than 30 LNs is better (31). However, achieving the goal of 30 ELNs for every patient remains a challenge. Certain factors have been reported to affect the ELN count, such as the extent of lymphadenectomy, tumor location, tumor size, the examiner’s technique or enthusiasm to find more LNs, and the innate number of LNs (10, 32). Some researchers believe that retrieving more LNs is an indicator of a high-quality operation but not the full outcome (33). Zhao et al. (34) reported that the prognoses of advanced-GC patients with ≥ 30 ELNs were not superior to those with 25–29 ELNs. The association between an increasing number of ELNs and improved OS outcomes was reported not to be necessarily causal (21, 33). However, the survival benefit of a greater number of ELNs continues to be achieved until a specific number or range of ELNs, from 10 to 29 across different studies (21, 35–37), representing the "glass ceiling" effect of ELNs on prognosis. Samer et al. (35) studied 40,281 GC patients and reported that the longest median survival was achieved at 29 dissected LNs; however, more than 29 LNs did not increase the median survival time, and dissection of ≥ 15 LNs was adequate for staging.

In our study, we found that 21 was the optimal cutoff value for ELNs to stratify N3 GC patients. To address the stage migration caused by the ELN count, we integrated ELNs (cutoff point: 21) into the 8th edition of the TNM staging system to reclassify patients according to their different 5-year OS rates and proposed a new modified TNM staging system. In the LASSO–Cox regression analysis, the mTNM staging system, age, tumor size, adjuvant chemotherapy, and adjuvant radiotherapy were identified as important factors associated with survival outcomes. Furthermore, the mTNM staging system was identified as the most important variable in predicting survival in the RSF analysis. We subsequently constructed an mTNM-based nomogram that included the variables of the mTNM staging system, age, tumor size, adjuvant chemotherapy, and adjuvant radiotherapy to predict the 1-, 3-, and 5-year OS rates. Although the 8th edition of the TNM staging system (AJCC/UICC) has been mostly accepted as a standardized tool to predict prognosis and perform adjuvant treatment, stage migration and inaccurate survival prediction are obviously present (38). The performance of the 8th edition of the TNM staging system for prognosis was validated for the U.S. population via the National Cancer Database (NCDB) (8, 38). However, the characteristics of GC differ between Western and Eastern populations, and heterogeneity clearly exists (39). With respect to the discriminative ability of survival, the 8th edition of the TNM staging system was poorer than the mTNM staging system and mTNM-based nomogram. Owing to the inclusion of the ELN count, the mTNM partly eliminates the effect of stage migration and better discriminates the prognosis. Li et al. (30) demonstrated that node-negative (N0) GC patients with ≤ 15 ELNs should be staged as N1 because their OS rates are similar to those of stage N1 patients with > 15 ELNs. A multicenter study detected significant stage migration in some N3a patients with < 16 ELNs, who should be classified into the N3b stage to achieve accurate prognostic evaluation (40). Furthermore, the risk of misclassification was found in 47.1% of GC patients when fewer than 10 LNs were examined (36). For patients with insufficient ELNs (< 21), the mTNM staging system allocates them into a later stage, which is the key to avoiding stage migration. Therefore, in the calibration curves, the predicted 1-, 3-, and 5-year OS rates were very close to the actual 1-, 3-, and 5-year OS rates when the mTNM-based nomogram was used.

The 8th edition of the TNM staging system is an essential basis for performing adjuvant chemotherapy or chemoradiotherapy (31, 41). With a heavy metastatic node burden, the incidence of peritoneal and locoregional relapse in stage N3 GC patients is relatively high, and the prognoses of these patients are poorer than those of any patients in earlier stages, making adjuvant therapy indispensable after curative gastrectomy (11, 12, 23). Pachaury et al. (23) and Bhandare et al. (26) suggested that more radical lymphatic clearance, beyond D2 dissection combined with more aggressive chemoradiotherapy, should be performed to achieve a synergistic effect for a better prognosis. Adjuvant chemotherapy and radiotherapy were proven to be independent prognostic factors. According to our findings, patients with insufficient ELNs (≤ 21) had a greater possibility of being at a lower stage, likely leading to inappropriate postoperative treatment and low-intensity surveillance (2). Furthermore, inadequate lymphadenectomy is the main reason for an insufficient number of LNs, resulting in an adverse prognostic outcome because of the high prevalence of micrometastatic disease and circulating tumor cells (42). Adjuvant chemotherapy and radiotherapy are crucial for decreasing both locoregional and distant recurrences after curative gastrectomy (14, 15, 43, 44). DCA revealed that the net benefits of using the mTNM-based nomogram and mTNM staging system were greater than those of the 8th edition of the TNM staging system. A possible explanation is that the mTNM could better discriminate patients with poor prognoses, followed by more aggressive postoperative adjuvant treatment and more regular surveillance or follow-up.

GC has a peak incidence in patients aged 50–70 years (45). Clinicopathologic and molecular features, such as diffuse type and Borrmann type IV features, are reportedly distinguishable between younger and older GC patients and are more commonly associated with younger patients, whereas atrophic gastritis and intestinal metaplasia are more common in older patients (46, 47). Kulig et al. demonstrated that postoperative morbidity and mortality rates increased with age (48). Moreover, age is a simple predictor of survival in patients with GC and should be considered along with important clinicopathological variables, such as depth of invasion and lymph node metastasis (49). Gastric tumor size is closely linked to the depth of invasion, vascular invasion, neural invasion, and peritoneal metastasis. However, it is controversial whether tumor size is an independent variable for predicting the prognosis of GC patients. Importantly, different cutoffs for tumor size were used. Larger tumors are usually related to a greater degree of malignancy and worse biological behavior. Lu et al. (50) reported that the addition of tumor size (< 5 and ≥ 5 cm) can improve the accuracy of the TNM staging system in predicting survival in GC patients undergoing radical surgery. Saito et al. (51) analyzed 1,473 GC patients with a size cutoff of 8 cm and reported that the survival rates of patients with stages II, IIIA, and IIIB disease with smaller tumors were similar to those of patients with stages IIIA, IIIB, and IV disease with large tumors, respectively. Thus, tumor size may be a good indicator of prognosis. In our study, the mTNM-based nomogram included variables of age and tumor size, which further increased the prediction accuracy of the prediction model compared with the 8th edition of the TNM staging system.

This study has several limitations. First, although the included population was large, the patients were recruited over a long period from 2004–2020 in the training cohort, and surgical techniques and chemoradiotherapy developed rapidly during this time, which may have resulted in population heterogeneity. Second, the external validation patients were from a single center; thus, selection bias could not be avoided. Third, although neural invasion, vessel invasion, and Her-2 status may impact the prognosis, these variables were not analyzed due to limitations in data acquisition. In the future, recruiting more patients and analyzing more variables from multiple centers will be necessary to validate and improve the mTNM staging system and the prediction model.

## Conclusions

The optimal cutoff value of ELNs for N3 GC patients to stage was 21. The mTNM staging system and mTNM-based nomogram showed superior discriminative ability, predictive accuracy, and greater net benefit for overall survival outcomes.

## Data Availability

The original contributions presented in the study are included in the article/**Supplementary Material**. Further inquiries can be directed to the corresponding author.
