# Supplementary material for: Construction of a modified TNM staging system and prediction model based on examined lymph node counts for gastric cancer patients at pathological stage N3
Source: Front Oncol. 2025 Apr 3;15:1569736. doi: 10.3389/fonc.2025.1569736 (PMC12003143; doi:10.3389/fonc.2025.1569736)
Supplement: Supplementary file 1 [file Table1.docx]

**Supplementary Table 1 General characteristics of the training cohort grouped by ELN count**

| **Variables** | **ELNs≤21 (n = 1969)** | **ELNs>21 (n = 2322)** | **χ^2^** | ***P*** |
| --- | --- | --- | --- | --- |
| **Age, n (%)** |  |  | 1.678 | 0.195 |
| ≤60 | 811 (41.2) | 1,003 (43.2) |  |  |
| >60 | 1,158 (58.8) | 1,319 (56.8) |  |  |
| **Sex, n (%)** |  |  | 0.221 | 0.638 |
| Female | 740 (37.6) | 890 (38.3) |  |  |
| Male | 1,229 (62.4) | 1,432 (61.7) |  |  |
| **Race, n (%)** |  |  | 12.79 | 0.005 |
| W | 1,292 (65.6) | 1,451 (62.5) |  |  |
| B | 272 (13.8) | 288 (12.4) |  |  |
| AI | 18 (0.9) | 26 (1.1) |  |  |
| API | 387 (19.7) | 557 (24) |  |  |
| **Tumor site, n (%)** |  |  | 9.112 | 0.028 |
| cardia/fundus | 465 (23.6) | 492 (21.2) |  |  |
| Body | 181 (9.2) | 254 (10.9) |  |  |
| antrum/pylorus | 605 (30.7) | 670 (28.9) |  |  |
| Others | 718 (36.5) | 906 (39) |  |  |
| **Tumor size, n (%)** |  |  | 7.369 | 0.007 |
| <8 cm | 1,451 (73.7) | 1,623 (69.9) |  |  |
| ≥8 cm | 518 (26.3) | 699 (30.1) |  |  |
| **Grade, n (%)** |  |  | 9.842 | 0.02 |
| Well | 20 (1) | 19 (0.8) |  |  |
| Moderate | 268 (13.6) | 282 (12.1) |  |  |
| Poor | 1,610 (81.8) | 1,968 (84.8) |  |  |
| Undifferentiated | 71 (3.6) | 53 (2.3) |  |  |
| **Histology, n (%)** |  |  | 3.429 | 0.18 |
| Adenocarcinoma | 1,153 (58.6) | 1,296 (55.8) |  |  |
| SRCC | 574 (29.2) | 713 (30.7) |  |  |
| others | 242 (12.3) | 313 (13.5) |  |  |
| **Lauren’s type, n (%)** |  |  | 10.256 | 0.017 |
| Intestinal type | 194 (9.9) | 257 (11.1) |  |  |
| Diffuse type | 207 (10.5) | 279 (12) |  |  |
| Mixed type | 76 (3.9) | 121 (5.2) |  |  |
| Others | 1,492 (75.8) | 1,665 (71.7) |  |  |
| **Adjuvant chemotherapy, n (%)** |  |  | 15.293 | <0.001 |
| No | 541 (27.5) | 517 (22.3) |  |  |
| Yes | 1,428 (72.5) | 1,805 (77.7) |  |  |
| **Adjuvant radiotherapy, n (%)** |  |  | 16.845 | <0.001 |
| No | 954 (48.5) | 1272 (54.8) |  |  |
| Yes | 1,015 (51.5) | 1,050 (45.2) |  |  |
| **T stage, n (%)** |  |  | 11.179 | 0.025 |
| T1 | 41 (2.1) | 68 (2.9) |  |  |
| T2 | 82 (4.2) | 108 (4.7) |  |  |
| T3 | 856 (43.5) | 979 (42.2) |  |  |
| T4a | 753 (38.2) | 943 (40.6) |  |  |
| T4b | 237 (12) | 224 (9.6) |  |  |
| **N stage, n (%)** |  |  | 727.105 | <0.001 |
| N3a | 1,783 (90.6) | 1,223 (52.7) |  |  |
| N3b | 186 (9.4) | 1,099 (47.3) |  |  |
| **TNM stage, n (%)** |  |  | 401.964 | <0.001 |
| IIB | 40 (2) | 48 (2.1) |  |  |
| IIIA | 79 (4) | 76 (3.3) |  |  |
| IIIB | 1,460 (74.1) | 1,062 (45.7) |  |  |
| IIIC | 390 (19.8) | 1,136 (48.9) |  |  |

ELNs, Examined lymph nodes; W: White, B: Black, AI: American Indian, API: Asian or Pacific Islander; SRCC, Signet ring cell carcinoma
