# Supplementary material for: Construction of a modified TNM staging system and prediction model based on examined lymph node counts for gastric cancer patients at pathological stage N3
Source: Front Oncol. 2025 Apr 3;15:1569736. doi: 10.3389/fonc.2025.1569736 (PMC12003143; doi:10.3389/fonc.2025.1569736)
Supplement: Supplementary file 2 [file Table2.docx]

**Supplementary Table 2 General characteristics of the validation cohort grouped by ELN count**

| **Variables** | **ELNs≤21 (n = 189)** | **ELNs>21 (n = 378)** | **χ^2^** | ***P*** |
| --- | --- | --- | --- | --- |
| **Age, n (%)** |  |  | 0.107 | 0.744 |
| ≤60 | 92 (48.7) | 191 (50.5) |  |  |
| >60 | 97 (51.3) | 187 (49.5) |  |  |
| **Sex, n (%)** |  |  |  |  |
| Female | 51 (27.0) | 93 (24.6) | 0.262 | 0.609 |
| Male | 138 (73.0) | 285 (75.4) |  |  |
| **Race, n (%)** |  |  | NA | NA |
| W | 0 | 0 |  |  |
| B | 0 | 0 |  |  |
| AI | 0 | 0 |  |  |
| API | 189 (100.0) | 378 (100) |  |  |
| **Tumor site, n (%)** |  |  |  |  |
| cardia/fundus | 79 (41.8) | 158 (41.8) | 0.119 | 0.989 |
| Body | 22 (11.6) | 41 (10.8) |  |  |
| antrum/pylorus | 65 (34.4) | 134 (35.4) |  |  |
| Others | 23 (12.2) | 45 (11.9) |  |  |
| **Tumor size, n (%)** |  |  | 0.446 | 0.504 |
| <8 cm | 153 (81.0) | 316 (83.6) |  |  |
| ≥8 cm | 36 (19.0) | 62 (16.4) |  |  |
| **Grade, n (%)** |  |  | 0.002 | 0.966 |
| Well | 0 | 0 |  |  |
| Moderate | 28 (14.8) | 54 (14.3) |  |  |
| Poor | 161 (85.2) | 324 (85.7) |  |  |
| Undifferentiated | 0 | 0 |  |  |
| **Histology, n (%)** |  |  | 0.724 | 0.696 |
| Adenocarcinoma | 154 (81.5) | 300 (79.4) |  |  |
| SRCC | 30 (15.9) | 70 (18.5) |  |  |
| others | 5 (2.6) | 8 (2.1) |  |  |
| **Lauren’s type, n (%)** |  |  | 7.132 | 0.068 |
| Intestinal type | 32 (16.9) | 74 (19.6) |  |  |
| Diffuse type | 66 (34.9) | 115 (30.4) |  |  |
| Mixed type | 54 (28.6) | 83 (22.0) |  |  |
| Others | 37 (19.6) | 106 (28.0) |  |  |
| **Adjuvant chemotherapy, n (%)** |  |  | 0.174 | 0.676 |
| No | 38 (20.1) | 69 (18.3) |  |  |
| Yes | 151 (79.9) | 309 (81.7) |  |  |
| **Adjuvant radiotherapy, n (%)** |  |  | 1.542 | 0.214 |
| No | 186 (98.4) | 377 (99.7) |  |  |
| Yes | 3 (1.6) | 1 (0.3) |  |  |
| **T stage, n (%)** |  |  |  |  |
| T1 | 3 (1.6) | 3 (0.8) | 9.094 | 0.059 |
| T2 | 8 (4.2) | 31 (8.2) |  |  |
| T3 | 135 (71.4) | 283 (74.9) |  |  |
| T4a | 35 (18.5) | 42 (11.1) |  |  |
| T4b | 8 (4.2) | 19 (5.0) |  |  |
| **N stage, n (%)** |  |  | 36.69 | <0.001 |
| N3a | 160 (84.7) | 223 (59.0) |  |  |
| N3b | 29 (15.3) | 155 (41.0) |  |  |
| **TNM stage, n (%)** |  |  | 38.12 | <0.001 |
| IIB | 2 (1.1) | 2 (0.5) |  |  |
| IIIA | 7 (3.7) | 23 (6.1) |  |  |
| IIIB | 146 (77.2) | 193 (51.1) |  |  |
| IIIC | 34 (18.0) | 160 (42.3) |  |  |

ELNs, Examined lymph nodes; W: White, B: Black, AI: American Indian, API: Asian or Pacific Islander; SRCC, Signet ring cell carcinoma
